# Is aspirin associated with diabetic retinopathy? The Singapore Epidemiology of Eye Disease (SEED) study

**DOI:** 10.1371/journal.pone.0175966

**Published:** 2017-04-28

**Authors:** Yuan Shi, Yih-Chung Tham, Ning Cheung, Jacqueline Chua, Gavin Tan, Paul Mitchell, Jie Jin Wang, Yin Bun Cheung, Ching-Yu Cheng, Tien Yin Wong

**Affiliations:** 1 Singapore Eye Research Institute, Singapore National Eye Centre, Singapore, Singapore; 2 Department of Ophthalmology, Yong Loo Lin School of Medicine, National University of Singapore, Singapore, Singapore; 3 Centre for Vision Research, Department of Ophthalmology and Westmead Institute for Medical Research, University of Sydeney, Sydeney, Australia; 4 Duke-NUS Medical School, Singapore, Singapore; 5 Tampere Center for Child Health Research, University of Tampere and Tampere University Hospital, Tampere, Finland; Swinburne University of Technology, AUSTRALIA

## Abstract

**Background/Aims:**

To determine the association between aspirin use and diabetic retinopathy (DR) among persons with diabetes, in a population-based, cross-sectional study.

**Methods:**

Subjects with diabetes aged >40 years from the Singapore Epidemiology of Eye Diseases Study were enrolled in this study. Retinal photographs were graded for DR according to the modified Airlie House classification system. Vision threatening diabetic retinopathy (VTDR) was defined as the presence of severe non-proliferative DR, or proliferative DR, or clinically significant macular oedema. The association between aspirin use and the presence of DR or VTDR was assessed using multivariable logistic regression models including age, gender, ethnicity, socioeconomic status, HbA1c, systolic blood pressure, anti-hypertension medicine, total cholesterol, anti-cholesterol medicine, BMI, current smoking status, diabetes duration, history of cardiovascular disease (CVD) and chronic kidney disease (CKD.).

**Results:**

A total of 2,061 participants with diabetes and complete record of relevant systemic and DR data were included. Of these, 711 (34.5%) had any stage of DR, and among these 177 (8.6%) had VTDR. After adjusting for co-variables listed, the association between aspirin use and VTDR was significant (OR = 1.69, *P* = 0.019), while the association between aspirin use and any DR was borderline (OR = 1.31, *P* = 0.063). Aspirin use was not associated with either DR or VTDR after additional adjustment of CVD and CKD. Further stratification by history of CVD or CKD showed no association between aspirin use and DR/VTDR in either subgroup.

**Conclusion:**

Aspirin use was not significantly associated with DR but might be an indicator of diabetic complications (CVD, CKD) that were co-present with more severe DR type. Future longitudinal studies are warranted to confirm our findings.

## Introduction

Diabetic retinopathy (DR) is a common microvascular complication of diabetes and is the leading cause of preventable vision loss among adult populations worldwide.[1] Globally, 34.6% persons with diabetes have DR. Of these, 10.2% have vision-threatening DR (VTDR). The number of persons with DR worldwide is expected to reach close to 200 million by 2030.[2,3]

The long-term vascular complication of diabetes with poor control include both macrovascular and microvascular complication.[4] Persons with diabetic macrovascular complications (e.g. CVD) are also more likely to have microvascular complications (e.g. DR). Aspirin is commonly used as a primary or secondary prevention to reduce the risk of CVD events and its related mortality[5,6]. While some investigators reported strong association between aspirin use and higher DR incidence[7], others showed that aspirin alone was not associated with risk of DR-related retinal or vitreous hemorrhage[8,9], and that aspirin use may even slow down progression of DR[10]. Previously observed association between aspirin use and DR by other investigators[7] could be the result of confounding by CVD condition, a concern that has not been examined thoroughly previously.

Hence, in the Singapore Epidemiology of Eye Disease (SEED) study, we sought to examine the association between aspirin use and DR in a large, multi-ethnic Asian population, taking into association account of the confounding effect by CVD. Better elucidation on this association may provide useful information for clinical management of diabetes.

## Materials and methods

### Study population

The subjects for this study were enrolled from the SEED, a population-based cross-sectional study of eye diseases in multi-ethnic groups of residents in Singapore aged 40 years and above. Briefly, an age-stratified random sampling of all Malay, Indian and Chinese adults residing in the southwestern part of Singapore was performed. A potential participant was considered to be ineligible if the person had moved from the residential address, had not lived there in the past 6 months, was deceased, or was terminally ill. Participation rate was calculated as the ratio of final participants by total eligible in each ethnic group. A total of 3,353 Chinese, 3,280 Malays, and 3,400 Indians participated in our study, giving response rates of 72.8%, 78.7%, and 75.6%, respectively. The detailed methodology of the SEED was described in previous publication.[11,12] The study adhered to the Declaration of Helsinki, and ethics approval was obtained from the Singapore Eye Research Institute (SERI)’s Institutional Review Board with written informed consent obtained from all subjects before participation. All participants underwent standardised ocular and systemic examination.

### Retinal photography and diabetic retinopathy assessment

DR was assessed through standardised retinal photography, using a digital retinal camera (Canon CR-DGi with 10D SLR back, Japan). After pupil dilation, two retinal photographs, centred at the optic disc and macula, were taken from both eyes. Photographs were graded at the University of Sydney by one certified ophthalmic grader, with adjudication by a senior retinal specialist. DR was considered present if characteristic lesions as defined by the Early Treatment Diabetic Retinopathy Study (ETDRS) (i.e. microaneurysms, haemorrhages, cotton wool spots, intraretinal microvascular abnormalities, hard exudates, venous beading, new vessels) were observed.[13,14] DR severity was graded based on the modified Airlie House classification system, using the Blue Mountains Eye Study protocol.[15,16] Individuals’ DR status was defined based on the severity scores of the worse eye. Clinically significant macular edema (CSME) was considered present when the macular edema involved was within 500 μm of the foveal center or if focal photocoagulation scars were present in the macular area. VTDR was defined as the presence of severe non-proliferative DR, proliferative DR or CSME.[17] The detailed protocol of DR grading was described in previous publication.[18]

### Clinical examination, questionnaire and interview

Comprehensive physical examination, laboratory tests and interview were performed as described elsewhere.[12,19] In brief, blood pressure (BP) was measured using a digital automatic blood pressure monitor after 5 minutes of rest. Body mass index (BMI) was calculated as body weight (kg) divided by body height (m) squared. Blood samples were collected to determine levels of serum lipids, glycated haemoglobin (HbA1c) and random glucose without fasting. Patients with diabetes were defined as having random glucose ≥11.1 mmol/L, use of diabetic medication or a participant-reported physician diagnosis of diabetes. Patients with hypertension were defined as systolic BP ≥ 140 mmHg, or diastolic BP ≥ 90 mmHg or physician diagnosis or self-reported history of hypertension. Patients with hyperlipidaemia were defined as total cholesterol ≥ 6.2 mmol/L or use of lipid lowering drugs.

A detailed interview was administered using a standardized questionnaire to collect information including medical history, duration of diabetes, educational level and monthly income. Aspirin use was defined as current intake of aspirin-type medication, including solprin, cardiprin, disprin, and ecotrin, but not panadol or dymadon. Use of anti-hypertensive drugs was defined as current intake of either ACE-inhibitors, angiotensin II receptor blocker, calcium channel blockers, diuretics, alpha receptor antagonists, beta receptor blockers or other anti-hypertensive medication not specified. Use of lipid lowering drugs was defined as current intake of either statins, fibrates, dyslipidaemic drugs or other anti-cholesterol medication not specified. CVD history was defined as a self-reported history or physician diagnosis of angina, or heart attack, or stroke. Chronic kidney disease (CKD) was defined as an estimated glomerular filtration rate (eGFR) <60 mL/min/1.73 m^2^, using the US National Kidney Foundation Kidney Disease Outcome Quality Initiative (KDOQI) Working Group definition.[20] eGFR was estimated from the serum creatinine concentration using the CKD Epidemiology Collaboration (CKD-EPI) equation[21]. As Socioeconomic status has been previously shown to be significantly associated with DR status[22,23], we included socioeconomic status as one potential confounder. Low socioeconomic status (SES) was defined as having primary or lower education, individual monthly income < SGD2000, and 1–2 room HDB flat.

### Statistical analysis

Statistical analysis was performed using R software version 3.2.2.[24] Baseline characteristics differences were tested using independent t-test for continuous variables and Pearson’s χ^2^ test for categorical variables. The association between aspirin use (exposure variable) and the presence of DR (outcome variable) was assessed using logistic regression models, adjusting for age, gender and ethnicity (Model 1), plus SES, HbA1c, blood pressure, cholesterol level, anti-hypertension drugs, anti-cholesterol drugs, BMI and smoking status (Model 2). We further adjusted for diabetes duration (Model 3) and additionally histories of CVD and CKD (Model 4). In all the statistical analysis, a *P* value of <0.05 is considered statistical significant. In the logistic regression models, odds ratios (ORs) and 95% confidence intervals (CIs) of the odds ratios are estimates generated to represent the associations between the study factor (aspirin use) with the outcome factor (DR), while controlling for co-variables that are potential confounding factors for the association under investigation.

## Results

Of the whole 10,033 SEED study participants, 2,888 participants had diabetes. After excluding those with unavailable data on DR grading, clinical information and social status, 2,061 participants were included in the final analysis. Among whom, 711 (34.5%) had any DR, 177 (8.6%) had VTDR, and 83 (4.0%) had CSME. Table 1 showed the demographic and clinical characteristic comparisons between the participants with and without DR. Among Chinese participants with diabetes, the proportion with DR (28%) was relatively lower than that among Malays or Indians (both around 36%) who had diabetes. Besides, the participants with DR are more likely to have hypertension, higher HbA1c, longer duration of diabetes, CVD history, CKD history, lower SES and to use aspirin. There were no significant differences in age, gender, cholesterol, presence of hyperlipidemia between participants with and without DR.

**Table 1:** Clinical characteristics comparison between diabetic patients with and without diabetic retinopathy (DR).

	Without DR (n = 1350)	With DR (n = 711)	*P*-value*
Age	61.7 (9.9)	61.9 (8.9)	0.753
Gender, Male	681 (50.44)	358 (50.35)	1.000
Ethnicity			
Chinese	305 (22.59)	121 (17.02)	**0.004**
Malay	437 (32.37)	242 (34.04)	0.474
Indian	608 (45.04)	348 (48.95)	0.100
Body Mass Index, kg/m^2^	26.91 (4.76)	26.34 (4.68)	**0.009**
Systolic blood pressure, mmHg	142.48 (20.44)	149.00 (23.26)	**<0.001**
Diastolic blood pressure, mmHg	77.54 (9.67)	77.49 (10.96)	0.921
Anti-hypertension medication use	786 (58.22)	442 (62.17)	0.092
Total cholesterol, mmol/L	5.06 (1.20)	5.04 (1.32)	0.806
Anti-cholesterol medication use	674 (49.93)	341 (47.96)	0.423
HbA1c, %	7.70 (1.73)	8.32(1.87)	**<0.001**
Duration of diabetes, years	8.4 (8.0)	13.5 (9.4)	**<0.001**
Hypertension	1015 (75.19)	588 (82.70)	**<0.001**
Hyperlipidaemia	846 (62.67)	426 (59.92)	0.241
Current smoking	183 (13.56)	82 (11.53)	0.217
Low socioeconomic status ^Τ^	88 (6.52)	63 (8.86)	0.064
History of cardiovascular disease	242 (17.93)	156 (21.94)	**0.033**
History of kidney disease	244 (18.07)	208 (29.25)	**<0.001**
Aspirin use	212 (15.70)	141 (19.83)	**0.021**

Data presented are means (standard deviation) or number (%), as appropriate for variables.

* *P*-value was obtained with t-test for continuous variables and with chi-square tests for categorical variables.

^Τ^ Low socioeconomic status was defined as Primary or lower education, Individual monthly income < SGD2000, and 1–2 room HDB flat.

After adjusting for potential confounders including age, gender, ethnicity, SES, HbA1c, systolic BP, anti-hypertensive medication, total cholesterol, anti-cholesterol medication, BMI and current smoking status, aspirin use was significantly associated with DR (OR = 1.35, 95% CI: 1.03 to 1.75, *P* = 0.028, Table 2, S1 Fig) and VTDR (OR = 1.89, 95% CI: 1.24 to 2.84, *P* = 0.003, Table 2), respectively. After further adjusting for diabetes duration, the association between aspirin use and VTDR remained significant (OR = 1.69, 95% CI: 1.09 to 2.61, *P* = 0.019, Table 2, S1 Fig), while the association between aspirin and DR was weaken (OR = 1.31, 95% CI: 0.99 to 1.74, *P* = 0.063, Table 2, S1 Fig). However, after additionally adjusted for history of CVD and CKD, aspirin use was no longer associated with DR (OR = 1.23, 95% CI: 0.90 to 1.67, *P* = 0.187, Table 2, S1 Fig) and VTDR (OR = 1.40, 95% CI: 0.86 2.25, *P* = 0.168, Table 2, S1 Fig). In comparison, the presence of CSME was not associated with aspirin use in any of the models above.

**Table 2:** Regression model showing the association between aspirin and DR.

	DR ^Τ^		VTDR ^Τ^		CSME ^Τ^	
	OR(95% CI)	*P*-value	OR(95% CI)	*P*-value	OR(95% CI)	*P*-value
**Model 1**	1.34 (1.05–1.71)	**0.018**	1.87 (1.27–2.71)	**0.001**	1.25 (0.67–2.20)	0.463
**Model 2**	1.35 (1.03–1.75)	**0.028**	1.89 (1.24–2.84)	**0.003**	1.52 (0.77–2.85)	0.206
**Model 3**	1.31 (0.99–1.74)	0.063	1.69 (1.09–2.61)	**0.019**	1.40 (0.71–2.67)	0.313
**Model 4**	1.23 (0.90–1.67)	0.187	1.40 (0.86–2.25)	0.168	1.16 (0.55–2.32)	0.691

Model 1: adjusted for age, gender and ethnicity.

Model 2: adjusted for variables in Model 1 plus socioeconomic status, HbA1c, systolic blood pressure, anti-hypertension medicine, total cholesterol, anti-cholesterol medicine, BMI, current smoking status.

Model 3: adjusted for variables in Model 2 plus duration of diabetes.

Model 4: adjusted for variables in Model 3 plus history of cardiovascular disease and chronic kidney disease

^Τ^ DR = diabetic retinopathy; VTDR = vision-threatening diabetic retinopathy; CSME = clinically significant macular edema; OR = odds ratio; CI = confidence interval.

The regression models were further stratified by history of CVD or CKD, respectively (Table 3, S2 Fig). The associations within each subgroup were not statistically significant. Nevertheless, we observed that the association between aspirin use and VTDR was slightly more prominent among individuals with CVD (OR = 1.99, 95% CI: 0.94 to 4.35, *P* = 0.076) compared to those without (OR = 1.10, 95% CI: 0.54 to 2.11, *P* = 0.790). No significant interaction effects were observed between CVD or CKD and aspirin.

**Table 3:** Regression model showing the association between aspirin use and VR or VTDR after stratified by history of cardiovascular disease or history of kidney disease.

	Stratification by CVD*				Stratification by CKD*			
	Without CVD		With CVD		Without CKD		With CKD	
	OR(95% CI)	*P*-value	OR(95% CI)	*P*-value	OR(95% CI)	*P*-value	OR(95% CI)	*P*-value
**DR**^Τ^	1.11 (0.74–1.66)	0.620	1.31 (0.80–2.17)	0.282	1.14 (0.78–1.66)	0.500	1.47 (0.85–2.54)	0.170
**VTDR**^Τ^	1.10 (0.54–2.11)	0.790	1.99 (0.94–4.35)	0.076	1.30 (0.62–2.60)	0.476	1.60 (0.81–3.16)	0.174

^Τ^ Both models adjusted for age, gender, ethnicity, socioeconomic status, HbA1c, systolic blood pressure, anti-hypertension medication, total cholesterol, anti-cholesterol medication, BMI, current smoking status, and duration of diabetes plus CKD in analysis of subgroup with CVD, and plus CVD in analysis of subgroup with CKD.

* CVD = cardiovascular disease; CKD = chronic kidney disease; OR = odds ratio; CI = confidence interval.

## Discussion

In this population-based study of a multi-ethnic Asian population, we examined the association of aspirin use and DR among persons with diabetes through involving CVD and CKD as potential confounders. Our results showed that persons with diabetes on regular aspirin use were more likely to have DR, especially VTDR. Nevertheless, this association became non-significant after further adjustment for history of CVD and CKD. This suggests that aspirin use is likely an indicator of CVD or CKD, both are complications of diabetes and likely co-present with DR at the relatively severe stage of diabetes.

Similar to the early part of our findings (Models 1 2 and 3 in Table 2), prospective data from the Madrid Diabetes Study of European cohort (MADIABETES) of 3,443 participants with diabetes, suggested that aspirin use was associated with 1.64-fold increased risk of 4-year incident DR after adjustment for gender, duration of diabetes, hypertension and HbA1c levels.[7] However, data from other studies generated conflicting results. For example, the Early Treatment for DR Study (ETDRS) showed that aspirin therapy was not associated with risk for DR progression.[13,14] In addition, the randomized clinical trial from the Dipyridamole Aspirin Microangiopathy of Diabetes (DAMAD) study group[10] and an earlier population study[8] showed that participants on aspirin had less retinopathy than those not on aspirin in a general diabetic population, suggesting that aspirin could be considered as an intervention to reduce risk of DR. These inconsistent results could have been due to differences in study population and designs. Of note, as an interventional clinical trial, there is more homogeneous health condition as ETDRS recruited patients with more severe DR, specifically CSME, and the DAMAD involved only participants with predominantly early DR. In contrast, MADIABETES enlisted participants with varied diabetes severities, while the history of previous but not CVD and CKD was not considered.

Therefore, it is important that evaluation in this area sufficiently takes into account the relevant confounding factors which are related to aspirin intake. In this regard, after further adjusting for history of CVD and CKD, our data showed that the associations between aspirin use and DR (any DR or VTDR) was attenuated and became non-significant (Model 4, Table 2). Further analyses stratified by CKD and CVD, respectively showed that the associations between aspirin use with DR and VTDR were more prominent in participants with previous history of either CVD or CKD than those without. Taken together, this may indicate that the initially observed associations of aspirin use with the presence of DR or VTDR might be the result of confounding by CVD and CKD, which are known diabetic complications at relatively advanced stage of diabetes, and also are indications of aspirin prescription.[25] The apparent association between aspirin and DR may merely be a reflection of its association with more severe diabetes, and may be confounded by indication of aspirin in individuals with CVD and CKD. We also included insulin in our models, and the conclusions remain the same (S1 Table).

Strengths of this study are the large, contemporary, population-based, multi-ethnic Asian sample of participants with diabetes, standardized assessment of DR based on high number of gradable photographs, and comprehensive evaluation of other relevant systemic factors and confounding variables. The results of this analysis should be interpreted after taking into account of the limitations. First, the cross-sectional design limited inference to causality or temporality of the reported association. Second, the lack of information on duration and dosage of aspirin use in our study also limited our analysis. Future studies with such information to determine if the relationship was dose-dependent.

In conclusion, our study demonstrated that an initially observed association between aspirin use and DR or VTDR became non-significant after further taking into account history of CVD and CKD, which are common complications of diabetes. This suggests that the previously reported elevated risk of DR associated with aspirin use is likely to be confounded by indication of aspirin use in individuals with CVD and CKD. Future longitudinal studies are warranted to confirm our findings, as this information has important clinical implication to the management of diabetes.

## Supporting information

S1 Fig Plot of association between aspirin use and DR status with odds ratio (OR) and 95% confidence interval (CI). The figure is consistent with the numbers in Table 3 in the main manuscript. The lines are color-coded according to outcome. Models with DR, VTDR and CSME as outcomes are plotted with blue, red and green, respectively. Model 1–4 are defined as in the main manuscript as below. Model 1: adjusted for age, gender and ethnicity. Model 2: adjusted for variables in Model 1 plus socioeconomic status, HbA1c, systolic blood pressure, anti-hypertension medicine, total cholesterol, anti-cholesterol medicine, BMI, current smoking status. Model 3: adjusted for variables in Model 2 plus duration of diabetes. Model 4: adjusted for variables in Model 3 plus history of cardiovascular disease and chronic kidney disease.
(TIFF)

S2 Fig Plot of associations between aspirin use and VR or VTDR status after stratified by history of cardiovascular disease or history of kidney disease, corresponding to Table 3 in the main manuscript. Models with Any_DR or VTDR as outcomes are plotted with blue or red respectively. The results are generated according to Model 3 in Table 2 with adjustment for age, gender and ethnicity, socioeconomic, duration of diabetes plus CKD in analysis of subgroup with CVD, and plus CVD in analysis of subgroup with CKD.
(TIFF)

S1 Table The association between aspirin and DR through regression models. (DOCX)

## References

[1] Lee R, Wong TY, Sabanayagam C. Epidemiology of diabetic retinopathy, diabetic macular edema and related vision loss. Eye Vis. 2015;2: 17. 10.1186/s40662-015-0026-2 PMC4657234 26605370

[2] Yau JW, Rogers SL, Kawasaki R, Lamoureux EL, Kowalski JW, Bek T, et al Global prevalence and major risk factors of diabetic retinopathy. Diabetes Care. 2012;35: 556–564. 10.2337/dc11-1909 22301125 PMC3322721

[3] Congdon N, Zheng Y, He M. The worldwide epidemic of diabetic retinopathy. Indian J Ophthalmol. 2012;60: 428 10.4103/0301-4738.100542 22944754 PMC3491270

[4] Fowler MJ. Microvascular and Macrovascular Complications of Diabetes. Clin Diabetes. 2008;26: 77–82.

[5] Colwell JA. Aspirin Therapy in Diabetes. Diabetes Care. 1997;20: 1767–1771. 9353620 10.2337/diacare.20.11.1767

[6] Mohamed Q, Gillies MC, Wong TY. Management of Diabetic Retinopathy. JAMA. 2007;298: 902 10.1001/jama.298.8.902 17712074

[7] Salinero-Fort MÁ, San Andrés-Rebollo FJ, de Burgos-Lunar C, Arrieta-Blanco FJ, Gómez-Campelo P. Four-Year Incidence of Diabetic Retinopathy in a Spanish Cohort: The MADIABETES Study. PLoS One. 2013;17: 10. 10.1371/journal.pone.0076417 PMC3798464 24146865

[8] Klein BEK, Klein R, Moss SE. Is Aspirin Usage Associated with Diabetic Retinopathy. Diabetes Care. 1987;10: 600–603. 3677978 10.2337/diacare.10.5.600

[9] Chew E, Klein M, RP M, Remaley N, Ferris F. Effects of aspirin on vitreous/preretinal hemorrhage in patients with diabetes mellitus. Early Treatment Diabetic Retinopathy Study report no. 20. Archives of Ophthalmology. 1995. 10.1001/archopht.1995.01100010054020 7826294

[10] The DAMAD Study Group. Effect of aspirin alone and aspirin plus dipyridamole in early diabetic retinopathy. A multicenter randomized controlled clinical trial. Diabetes. 1989;38: 491–498. 2647556

[11] Lavanya R, Jeganathan VSE, Zheng Y, Raju P, Cheung N, Tai ES, et al Methodology of the Singapore Indian Chinese Cohort (SICC) eye study: quantifying ethnic variations in the epidemiology of eye diseases in Asians. Ophthalmic Epidemiol. 2009;16: 325–336. 10.3109/09286580903144738 19995197

[12] Wong TY, Chong EW, Wong W-L, Rosman M, Aung T, Loo J-L, et al Prevalence and causes of low vision and blindness in an urban malay population: the Singapore Malay Eye Study. Arch Ophthalmol. 2008;126: 1091–1099. 10.1001/archopht.126.8.1091 18695104

[13] Early Treatment Diabetic Retinopathy Study Research Group. Fundus photographic risk factors for progression of diabetic retinopathy. ETDRS report number 12. Ophthalmology. 1991;98: 823–833. 2062515

[14] Group ETDRSR. Early Treatment Diabetic Retinopathy Study design and baseline patient characteristics. ETDRS report number 7. Ophthalmology. 1991;98: 741–756. 2062510 10.1016/s0161-6420(13)38009-9

[15] Cikamatana L, Mitchell P, Rochtchina E, Foran S, Wang JJ. Five-year incidence and progression of diabetic retinopathy in a defined older population: the Blue Mountains Eye Study. Eye. 2007;21: 465–471. 10.1038/sj.eye.6702771 17318200

[16] Mitchell P, Smith W, Wang JJ, Attebo K. Prevalence of diabetic retinopathy in an older community: The blue mountains eye study. Ophthalmology. 1998;105: 406–411. 10.1016/S0161-6420(98)93019-6 9499768

[17] Kempen JH, O’Colmain BJ, Leske MC, Haffner SM, Klein R, Moss SE, et al The prevalence of diabetic retinopathy among adults in the United States. JAMA Epidemiol. 2004;122: 552–563. 10.1001/archopht.122.4.552 15078674

[18] Wong TY, Cheung N, Tay WT, Wang JJ, Aung T, Saw SM, et al Prevalence and Risk Factors for Diabetic Retinopathy. The Singapore Malay Eye Study. Ophthalmology. 2008;115: 1869–1875. 10.1016/j.ophtha.2008.05.014 18584872

[19] Pan CW, Wong TY, Chang L, Lin XY, Lavanya R, Zheng YF, et al Ocular biometry in an Urban Indian population: The Singapore Indian Eye study (SINDI). Investig Ophthalmol Vis Sci. 2011;52: 6636–6642. 21791589 10.1167/iovs.10-7148

[20] National Kidney Foundation. K/DOQI clinical practice guidelines for chronic kidney disease: evaluation, classification, and stratification. Am J Kidney Dis. 2002;39: 1–266. 11904577

[21] Levey AS, Stevens LA, Schmid CH, Zhang YL, Castro AF, Feldman HI, et al A new equation to estimate glomerular filtration rate. Ann Intern Med. 2009;150: 604–612. 19414839 10.7326/0003-4819-150-9-200905050-00006 PMC2763564

[22] Raman R, Rani PK, Reddi Rachepalle S, Gnanamoorthy P, Uthra S, Kumaramanickavel G, et al Prevalence of diabetic retinopathy in India: Sankara Nethralaya Diabetic Retinopathy Epidemiology and Molecular Genetics Study report 2. Ophthalmology. 2009;116: 311–318. 10.1016/j.ophtha.2008.09.010 19084275

[23] Scanlon PH, Carter SC, Foy C, Husband RFA, Abbas J, Bachmann MO. Diabetic retinopathy and socioeconomic deprivation in Gloucestershire. J Med Screen. 2008;15: 118–121. 10.1258/jms.2008.008013 18927093

[24] Venables WN, Smith DM. R Development Core Team. An Introduction to R Notes on R A Programming Environment for Data Analysis and Graphics R core team version 2008.

[25] Valmadrid CT, Klein R, Moss SE, Klein BE. The Risk of Cardiovascular Disease Mortality Associated with Microalbuminuria and Gross Proteinuria in Persons with Older-Onset Diabetes Mellitus. JAMA Intern Med. 2000;160: 1093–1100. 10.1001/archinte.160.8.1093 10789601

