# Supplementary figures and images for: Is aspirin associated with diabetic retinopathy? The Singapore Epidemiology of Eye Disease (SEED) study
Source: PLoS One. 2017 Apr 28;12(4):e0175966. doi: 10.1371/journal.pone.0175966 (PMC5409055; doi:10.1371/journal.pone.0175966)

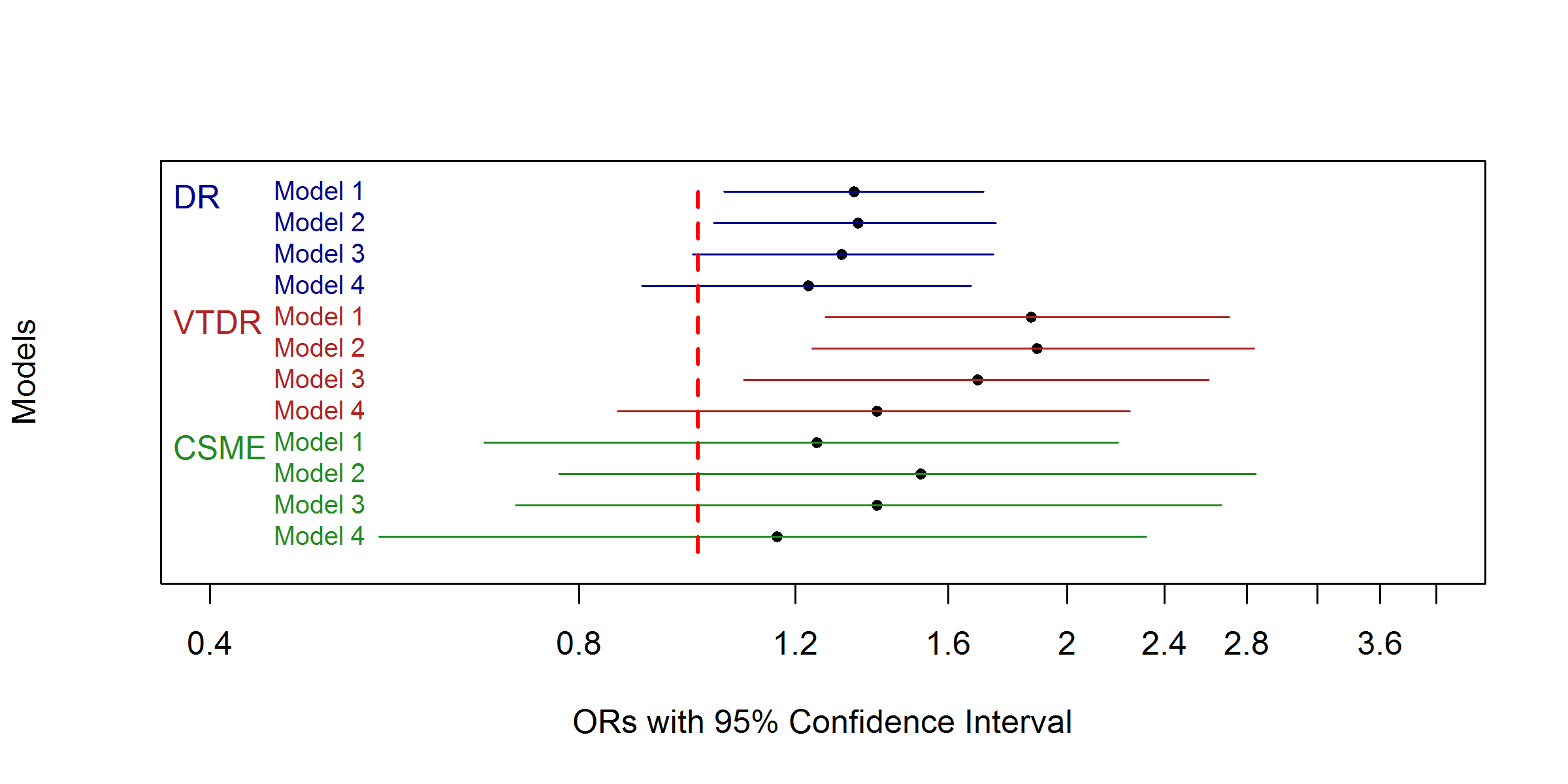

Supplement: S1 Fig — The figure is consistent with the numbers in Table 3 in the main manuscript. The lines are color-coded according to outcome. Models with DR, VTDR and CSME as outcomes are plotted with blue, red and green, respectively. Model 1–4 are defined as in the main manuscript as below. Model 1: adjusted for age, gender and ethnicity. Model 2: adjusted for variables in Model 1 plus socioeconomic status, HbA1c, systolic blood pressure, anti-hypertension medicine, total cholesterol, anti-cholesterol medicine, BMI, current smoking status. Model 3: adjusted for variables in Model 2 plus duration of diabetes. Model 4: adjusted for variables in Model 3 plus history of cardiovascular disease and chronic kidney disease. (TIFF) [file pone.0175966.s001.tiff]

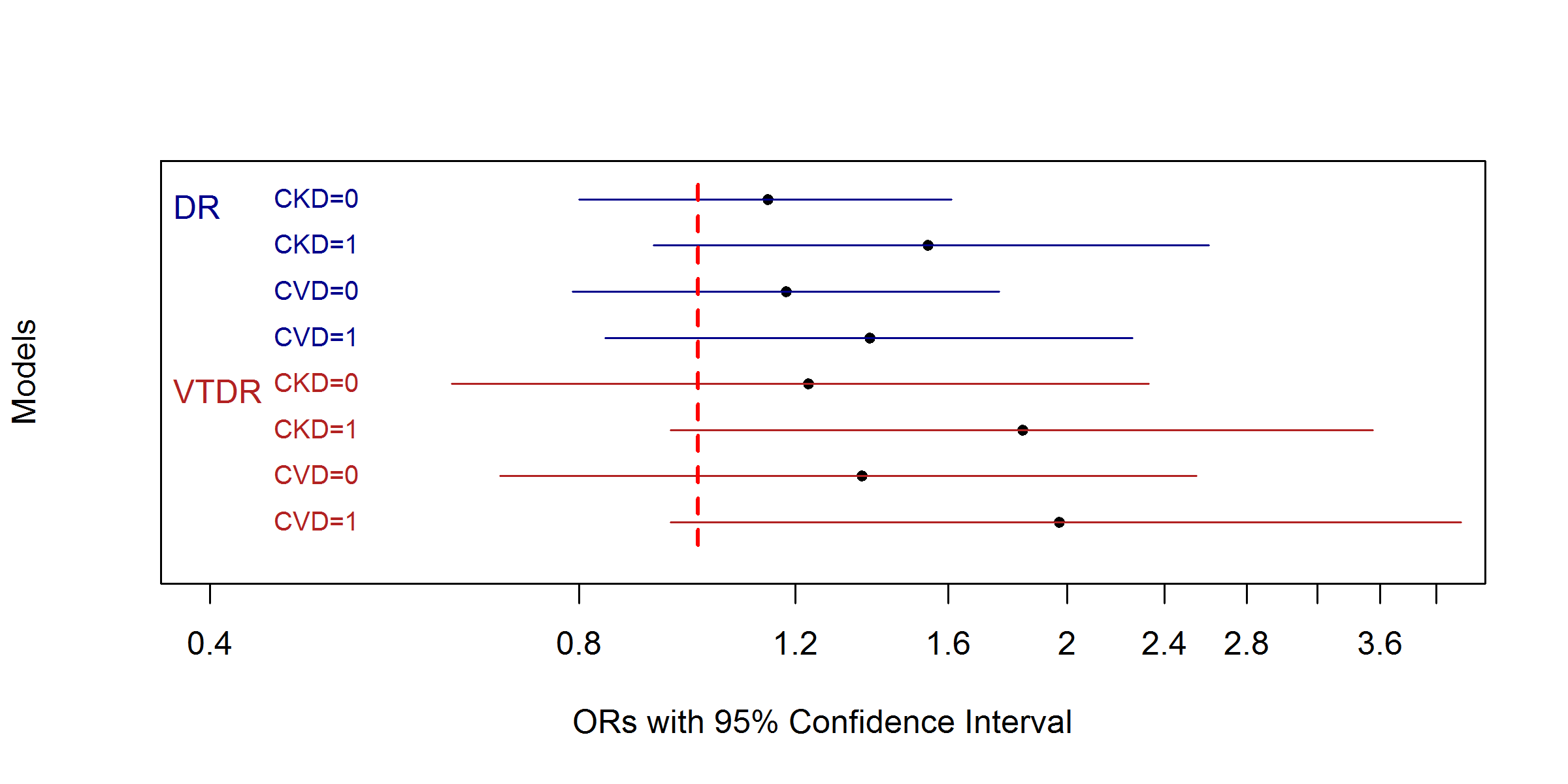

Supplement: S2 Fig — Models with Any_DR or VTDR as outcomes are plotted with blue or red respectively. The results are generated according to Model 3 in Table 2 with adjustment for age, gender and ethnicity, socioeconomic, duration of diabetes plus CKD in analysis of subgroup with CVD, and plus CVD in analysis of subgroup with CKD. (TIFF) [file pone.0175966.s002.tiff]
