# Supplementary material for: Is aspirin associated with diabetic retinopathy? The Singapore Epidemiology of Eye Disease (SEED) study
Source: PLoS One. 2017 Apr 28;12(4):e0175966. doi: 10.1371/journal.pone.0175966 (PMC5409055; doi:10.1371/journal.pone.0175966)
Supplement: S1 Table — (DOCX) [file pone.0175966.s003.docx]

**S1 Table. The association between Aspirin and DR status through regression models**

|  | **DR** ^Τ^ | |  | **VTDR** ^Τ^ | |  | **CSME** ^Τ^ | |
| --- | --- | --- | --- | --- | --- | --- | --- | --- |
|  | **OR(95% CI)** | ***P*-value** |  | **OR(95% CI)** | ***P*-value** |  | **OR(95% CI)** | ***P*-value** |
| Model-1 | 1.22 (0.95-1.58) | 0.122 |  | 1.57 (1.05-2.32) | 0.026 |  | 1.04 (0.55-1.86) | 0.906 |
| Model-2 | 1.24 (0.94-1.63) | 0.118 |  | 1.62 (1.05-2.48) | 0.028 |  | 1.29 (0.65-2.45) | 0.452 |
| Model-3 | 1.25 (0.94-1.68) | 0.128 |  | 1.55 (0.99-2.42) | 0.054 |  | 1.26 (0.63-2.43) | 0.498 |
| Model-4 | 1.18 (0.86-1.61) | 0.311 |  | 1.27 (0.77-2.06) | 0.338 |  | 1.05 (0.5-2.12) | 0.898 |

Model 1: adjusted for age, gender, ethnicity and ***Insulin.***

Model 2: adjusted for variables in Model 1 plus socioeconomic status, HbA1c, systolic blood pressure, anti-hypertension medicine, total cholesterol, anti-cholesterol medicine, BMI, current smoking status.

Model 3: adjusted for variables in Model 2 plus duration of diabetes.

Model 4: adjusted for variables in Model 3 plus history of cardiovascular disease and chronic kidney disease

^Τ^DR = diabetic retinopathy; VTDR = vision-threatening diabetic retinopathy; CSME = clinically significant macular edema; OR= odds ratio; CI= confidence interval.
